# Safetyome and specialized panels for over 3,000 phenotypes: a systematic and translational approach using human genetics and pharmacology

**DOI:** 10.1093/toxsci/kfag021

**Published:** 2026-02-19

**Authors:** Xin Liu, Yen-Wei Chen, Xiao Xu, David Smith, Fan Fan

**Affiliations:** Preclinical Sciences & Translational Safety, Johnson & Johnson, 3210 Merryfield Rd, San Diego, CA 92121, USA; Preclinical Sciences & Translational Safety, Johnson & Johnson, 3210 Merryfield Rd, San Diego, CA 92121, USA; In silico Discovery, Johnson & Johnson, 1400 McKean Rd, Spring House, PA 19477, USA; Preclinical Sciences & Translational Safety, Johnson & Johnson, 3210 Merryfield Rd, San Diego, CA 92121, USA; Preclinical Sciences & Translational Safety, Johnson & Johnson, 3210 Merryfield Rd, San Diego, CA 92121, USA

**Keywords:** translational sciences, systems biology, hazard identification, omics research, organ toxicity, 3,000+ specialized panels

## Abstract

Drug candidates are often evaluated for their activities against unexpected targets (off-targets), to either prospectively flag potential hazards or to provide mechanistic insights for a given phenotype. The *in vitro* to *in vivo* translatability is critical when selecting which “phenotypically consequential” off-targets to screen. To this end, human genetics and indication-based pharmacology offer unraveled insights. Enhanced natural language processing tools were applied to harness the power of large data obtained from 7 genetics and 2 pharmacology databases. Mapping biological roles to organ systems, we curated targets implicated in 22 organ systems of safety concerns, resulting in a safetyome composed of over ∼11,000 proteins. This is a significant expansion from our previously proposed screen, whose scope included phenotypes affecting 5 organ systems. Prioritization of the large panel using expression pattern and gene conservation across species resulted in a core panel of 500 targets. Mapping biological roles obtained from the databases to specific terms allowed us to systematically generate over 3,000 phenotype-based (specialized) panels, which can be used as gene or protein sets for issue resolution. All three components: The full safetyome, the core panel of 500 targets, and the over 3,000+ specialized panels, were systematically and orthogonally tested using independent data source, i.e., gene expression data from the Comparative Toxicogenomics Database. All panels, together with a user-friendly App, are published to aid effective safety assessment and issue resolution with strong “translational” focus.

**Abbreviations:** MedDRA: (Medical Dictionary for Regulatory Activities); NLP: (natural language processing); UMLS: (Unified Medical Language System); CTD: (Comparative Toxicogenomics Database); SOC: (system organ class); PT: (preferred term); GWAS: (Genome-wide association studies); HPO: (Human Phenotype Ontology)

## Introduction

Drug candidates undergo assessments at different stages to evaluate activities against unexpected targets (off-targets), from secondary pharmacology profiling to mechanistic investigations of toxicological findings (issue resolution), for which *in vitro* assays and various omics platforms, such as transcriptomics or proteomics, are used (Verbist et al. 2015; Whitebread et al. 2016; Jenkinson et al. 2020; Liu et al. 2021; Brennan et al. 2024). Secondary pharmacology profiling counter-screens drug candidates to identify hazard(s) prior to *in vivo* toxicological studies in preclinical species. The panel typically contains ∼70 to 200 off-targets, for which high-throughput *in vitro* assays are used (Bowes et al. 2012; Whitebread et al. 2016; Jenkinson et al. 2020; Brennan et al. 2024). The number of off-targets interrogated can increase exponentially with new therapeutics, such as RNA interference or protein degradation, for which genome- or proteome-wide screening is performed (Verbist et al. 2015; Liu et al. 2021). When an unexpected toxicological finding arises, investigative studies are often conducted with the goal of informing mechanism and/or mitigating human risks, in which case testing against a comprehensive panel of off-targets transcript or protein set may offer valuable mechanistic insights. These panels should contain causal targets for which *in vivo* to *in vitro* translatability are high, as interfering with a target might have little or no phenotypic manifestation due to the existence of redundant genes or compensatory pathways (Nowak et al. 1997; Gibson and Spring 1998; Gu et al. 2003; Vavouri et al. 2008; Laruson et al. 2020). To ensure high *in vitro* to *in vivo* translatability, current practices still need improvement, as the selection of relevant off-targets is manual and mostly based on literature, historical hit rates, legacy institutional experience, and assay feasibility (Bowes et al. 2012; Whitebread et al. 2016; Jenkinson et al. 2020; Brennan et al. 2024). While valuable, these factors do not adequately address translatability in safety assessments (Papoian et al. 2015; 2017), and is not suitable to compose larger omics-wide panel and assess (potentially more) off-targets in a cohesive fashion. As for specialized and phenotype-based panels used for issue resolution, the commercially available gene and protein sets do not cover the plethora of phenotypes encountered by pharmaceutical companies on a daily basis. Also, the manual off-target selection process and non-standardized data sources are highly variable, often resulting in inconsistent and incomplete gene or protein sets to allow fruitful mechanistic studies.

Human genetics, through Mendelian diseases and genome wide association studies and findings from genome wide association (GWAS) (Cerezo et al. 2025), provides direct insights to establish association or causal link between targets and phenotypes. Targets with genetic associations have higher likelihood to be phenotypically consequential (higher translatability), which was demonstrated by numerous studies (Nelson et al. 2015; Nguyen et al. 2019; Carss et al. 2023). Similarly, pharmacology, stemming from the pursuit of a given target therapeutics for specific indications, also affords the opportunity to establish a direct link between targets and phenotypes. Recent publications have shown that the integration of human genetics and indication-based pharmacology facilitates the rational nomination of off-targets that are likely to exhibit greater *in vitro in vivo* translatability (Deaton et al. 2019; Liu et al. 2021). These prior works focused solely on 5 organs: Cardiovascular, central nervous and respiratory systems, and comprise the “selected off-target proteome” (SOTP) which informs omics-based screening used in new modalities (Liu et al. 2021). In the present study, we deployed an improved language processing pipeline (S Pradeep 2024) and integrated 9 human genetics and pharmacology databases to compose a full “safetyome,” expanding areas of focus beyond what was previously available and representing 22 organs such as eye, kidney, skin, liver, endocrine and others as detailed in Table S1. Additional biological metrics, such as tissue expression in humans and genetic conservation across species, were used to prioritize the targets within the full safetyome, yielding a core panel containing 500 targets. By mapping the biological roles of these targets to standardized yet more specific phenotype terms, we systematically generated panels encompassing over 3,000 phenotypes. All three components: Safetyome full and core panels, as well as over 3,000 specialized phenotype-based panels, were tested using an orthogonal source, i.e., Comparative Toxicogenomics Database (CTD) (Davis et al. 2025). The full and core panels can be used during lead optimization for general hazard identification, while the specialized panels can be used during issue resolution where a particular phenotype is being investigated. Importantly, to aid the use across drug development and the entire scientific community with the goal of “making safer drugs,” a user-friendly PhenoSOC and PhenoPT App was deployed (https://jnjtst.shinyapps.io/safetyome_v1-1/).

## Materials and methods

### Database harmonization and mapping biological roles

Human genetics databases used were: GWAS catalog (Catalog; Cerezo et al. 2025), ClinVar (ClinGen; Henrie et al. 2018; Landrum et al. 2020; Zhang et al. 2013), Human Phenotype Ontology (HPO) (Gargano et al. 2024; Ontology), Phenotype-Genotype Integrator Phe-Genl (I; Ramos et al. 2014), DISEASES Disease-gene associations mined from literature database (Grissa et al. 2022), DisGeNET (Pinero et al. 2020), Open Targets (Buniello et al. 2025; Platform). The pharmacology databases used were DrugBank (DrugBank; Wishart et al. 2018; Knox et al. 2024) and the Therapeutic Target Database (TTD) (Database; Zhou et al. 2024).

After bulk downloading, filtering criteria were applied to exclude non-protein coding genes and refine associations based on statistical significance and relevance. For GWAS catalog and Phe-Genl, only associations exhibiting a *P*-values ≤ 5 × 10^−8^ were retained (International HapMap 2005; Pe’er et al. 2008). To further ensure the robustness and interpretability of the data in GWAS catalog, records containing invalid or missing odds ratios or beta coefficients were eliminated. For ClinVar, records with missing (“NA”) source annotations were excluded prior to downstream processing. After this step, the remaining ClinVar associations comprised MONDO, Human Phenotype Ontology (HPO), OMIM, OMIM phenotypic series, NCBI curation, Orphanet, and GeneReviews. For DisGeNET, we applied genetics-focused filters because the association-type ontology includes relationship classes beyond human genetics (e.g. biomarker, altered expression, post-translational modification). To align with the scope of the Safetyome, only association types reflecting genetic evidence were retained, including “CausalMutation,” “GermlineCausalMutation,” “SomaticCausalMutation,” “SusceptibilityMutation,” “ModifyingMutation,” and “GeneticVariation.” In addition, we also limited DisGeNET records to sources capturing human genetic evidence, including ClinGen, ClinVar, Genomics England, GWAS Catalog, GWASDB, HPO, and Orphanet. In terms of drug indications from DrugBank, we utilized both structured indications and those extracted through NLP using SciSpacy (Neumann et al. 2019).

The phenotypes and biological roles obtained from these databases were mapped according to the hierarchy outlined by Medical Dictionary for Regulatory Activities (MedDRA, MDR 26_1 release) (Activities; Fey et al. 2024) using a multi-step pipeline based on established tools. We first utilized the Unified Medical Language System (UMLS) (Bodenreider 2004) API (version 2024-01) to conduct both exact and partial mapping. For each phenotype, we retained the top-ranked concept. When a given mapped concept was associated with multiple PTs in the hierarchy, we retained all such PTs and their corresponding SOCs, rather than forcing an arbitrary single label. To increase recall for terms that did not map through this route, we next employed MetaMap (Kang et al. 2021) (strict version 2018) to enhance the mapping process, facilitated by the Python wrapper pymetamap (Rios 2022), again selecting the highest-ranked concept per phenotype. Finally, we adopted the pretrained PubMedBERT model (Gu et al. 2022) from the Hugging Face Transformers (Wolf et al. 2019) for mapping unmapped terms to the entire MedDRA hierarchy at a threshold of 0.7, a parameter shown to effectively balance accuracy and data retrieval in previous studies (Pradeep 2024). Two MedDRA hierarchical levels were used in our study for two different purposes, System Organ Class (SOC) at the organ level and Preferred Term (PT) at a more specific level to reflect a given phenotype or symptom. The SOC level mapping is to quickly flag out safety concerns to standardized terms. The PT level mapping allowed us to group targets based on phenotypes, hence systematically obtain large number of specialized panels. The full pipeline used to construct the SOC-level and PT-level safetyome has been implemented in a repository (https://github.com/sarahxin/safetyome-phenomapping-pipeline.git).

### Scoring methodology

To assess the relevance of each gene based on the occurrence across multiple sources, the raw score was computed using equation 1. To standardize the scores across targets, we applied *Z*-score normalization (equation 2), which was then log transformed to reduce the impact of extreme values (equation 3). For easier interpretation, a min-max rescaling using equation 4 was carried out to transform the transformed scores to a range of 0 to 10. All computations were performed using Python.


(1) $$\mathrm{Raw}\;\mathrm{Score}=(\sum_{i}^{n}{\mathrm{count}\;\mathrm{of}\;\mathrm{source}}_{i})\;\times \;\log_{e\;}\left(\mathrm{number}\;\mathrm{of}\;\mathrm{unique}\;\mathrm{sources}+1\right)$$

(2) $$Z_{\mathrm{score}}=\;\frac{\mathrm{raw}\;\mathrm{score}-\mu_{\mathrm{raw}\;\mathrm{score}}}{\sigma_{\mathrm{raw}\;\mathrm{score}}}\;$$

(3) $${\mathrm{Log}\;Z}_{\mathrm{score}}=\log_{e\;}(Z_{\mathrm{score}}-\min\left(Z_{\mathrm{score}})+1\right)\;$$

(4) $$\mathrm{Scaled}\;\mathrm{Score}=10\;\times \;\frac{{\mathrm{Log}\;Z}_{\mathrm{score}}-\min\left({\;\mathrm{Log}\;Z}_{\mathrm{score}}\right)}{\max\left({\mathrm{Log}\;Z}_{\mathrm{score}}\right)-\min({\mathrm{Log}\;Z}_{\mathrm{score}})}\;$$

### Orthogonal testing using comparative toxicogenomics database (CTD)

CTD (Database; Davis et al. 2025) that aggregates Gene–disease associations were used to as an orthogonal source to test our database. Disease genes associated with CTD were documented. Only genes with direct disease evidence were used. Subsequently, gene overlapping analysis from R clusterprofiler package, version 4.3.2 (Yu et al. 2012; Wu et al. 2021) was performed to identify enriched CTD disease phenotypes in safetyome genes on SOC or PT level. The enrich function from clusterprofiler package was applied to obtain over-representation statistics Significant enrichments were further extracted and computed for the percentage of matching disease phenotypes to SOC/PT level. For PT-level analyses, we restricted comparisons to safetyome PT panels containing ≥50 targets to reduce bias and instability associated with small target sets. We applied Bonferroni correction (Bonferroni 1936) on *P*-value to correct potential biases caused by testing large numbers of PT terms. For analysis with score cutoff from SOC level, targets from each SOC were further filtered based on their score percentile retrieved from previous session to determine if additional target information improves matching results. We then evaluated the alignment between NLP-mapped phenotypes at the SOC or PT levels and the enriched categories derived from gene set enrichment analysis. By analyzing match rates at various cutoff thresholds, we identified the threshold that achieved the optimal matches, effectively capturing biologically relevant gene-phenotype relationships while minimizing noise from weak or overly broad associations.

### Target prioritization

Safetyome targets were ranked based on two metrics, Tau index and conservation scores. Tau index obtained from Genotype-Tissue Expression (GTEx, v8) (Lonsdale et al. 2013; Lüleci and Yılmaz 2022) was used to rank overall tissue specificity in each target as published previously (Palmer et al. 2021). Conservation score was computed based on median of two sources: (i) the percentage of identity for the primary gene sequence between human and preclinical species (i.e. mouse, rat and dog); and (ii) whether the gene ortholog was confidently identified based on ENSEMBL. Conservation metrics from each source were ranked. The median across species first followed by averaging from two categories to represent conservation score.

## Results

### Target-trait associations and phenotype mapping

**Fig. 1.**
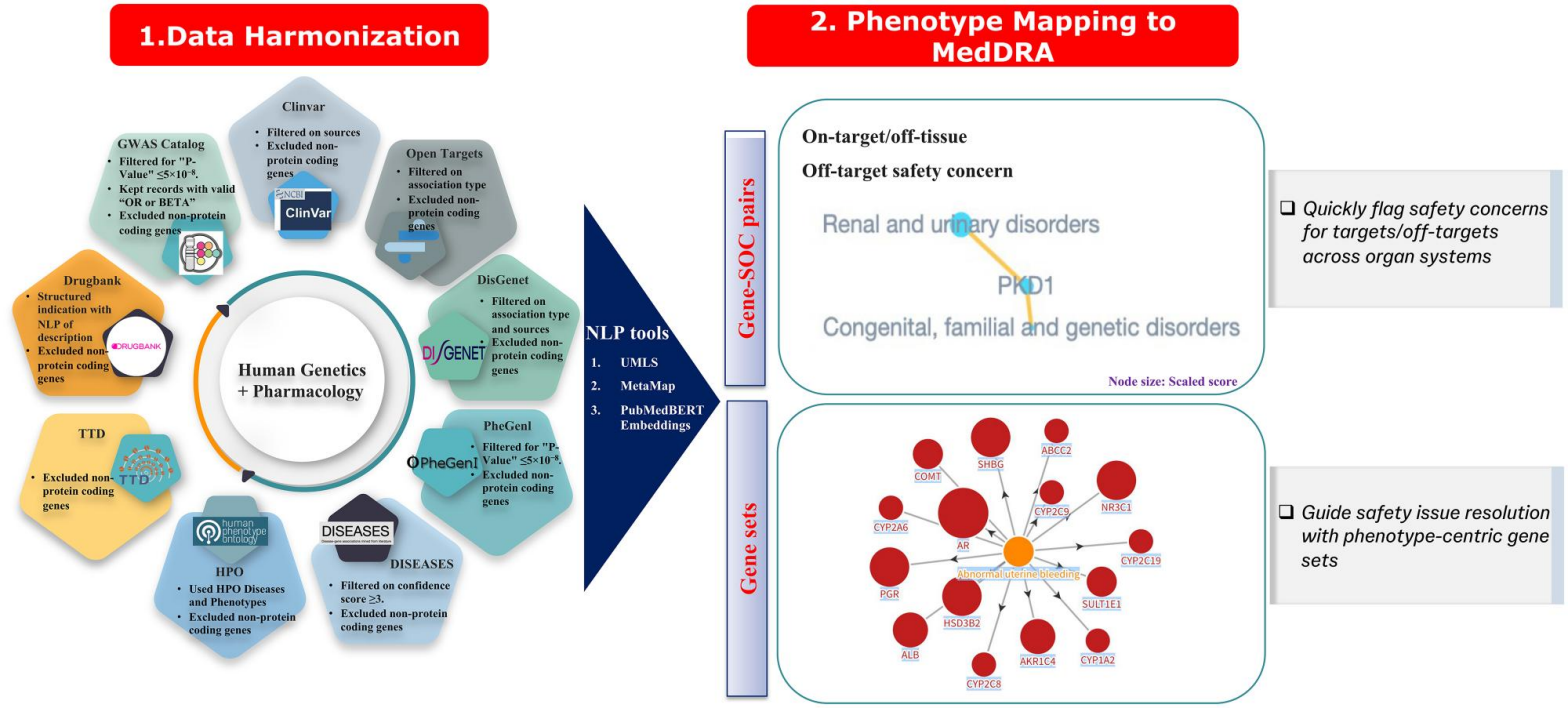
Workflow for data harmonization and phenotype mapping. Data harmonization encompasses the curation of genetic and pharmacological data from nine distinct sources. Filtering criteria were established to refine associations based on statistical significance and source relevance. Phenotype mapping to the hierarchy employs NLP tools to systematically map terms or text to the MedDRA classification.

In this study, we obtained a total of 18,356 targets and 63,074 traits by consolidating data from 7 genetic databases and 2 pharmacological databases as shown in the workflow in Fig. 1. This allowed the identification of 863,041 target-trait pairs. As illustrated in Fig. S1, the most extensive target coverage was derived from GWAS Catalog (Catalog; Cerezo et al. 2025) and Open Targets (Buniello et al. 2025; Platform), with HPO (Gargano et al. 2024; Ontology) also contributing significantly to gene-trait associations. Although some associations were reported across multiple sources, a notable proportion of target-trait links (97.9%) are unique to individual databases. This finding demonstrated the necessity and value of harmonizing diverse datasets into a standardized vocabulary to obtain maximal gene-trait pairs.

To evaluate the robustness and completeness of phenotype mapping, we examined the contributions of various Natural Language Processing (NLP) tools. Fig. S2A shows that the UMLS (Bodenreider 2004) serves as a strong baseline for mapping phenotypic descriptions to standardized terminologies, successfully accounting for 74.5% (47,005 terms) of the original terms. The sequential uses of MetaMap (Kang et al. 2021) and then embeddings (Gu et al. 2022) further increased coverage by 7.3% (4,626 terms) and 4.9% (3,113 terms), respectively, resulting in a total coverage of 86.8%, demonstrating the advantage of combined use of different NLP tools.

The proportion of unmapped terms with MedDRA (Activities; Fey et al. 2024) across various data sources was shown in Fig. S2B. The GWAS Catalog exhibits highest percentage of unmapped terms (27.3%), suggesting that GWAS-derived phenotypic descriptions may be beyond the scope and complexity defined by MedDRA. Similarly, unmapped terms in HPO (11.2%) and DisGeNet (9.8%) (Pinero et al. 2020) indicate that phenotype descriptions concerning rare diseases require improved standardization. The top 30 most frequently unmapped traits offer insights into specific terms where phenotype mapping remains incomplete, as depicted in Fig. S2C. Many of these traits involve complex, composite descriptions such as “hip circumference adjusted for BMI,” “educational attainment,” “absent speech” or “long face” that do not correspond to direct one-to-one matches within MedDRA. Among the targets associated with unmapped terms, only 60 were unique and not covered by mapped traits, indicating a limited impact on the overall results (Fig. S2D). The UpSet plots presented in Fig. S2F reveals that 45.4% of System Organ Class (SOC)-level associations are supported by multiple sources.

### Defining and testing the safetyome using comparative toxicogenomics database (CTD)

Five out of 27 SOC terms were eliminated as they cannot be accurately mapped to specific organs. The SOCs used are summarized in Table S1. The resulting 17,372 targets were further ranked based on the strength of evidence, to which a scoring method detailed in the Materials and Methods (Scoring Methodology) section was applied. The raw scores were calculated based on occurrences across various sources, with additional consideration for the number of supporting databases. This approach ensures that targets appearing in multiple sources receive higher scores, while preventing excessive weighting of targets that appear repeatedly in a single source. Following this, normalization to scaled scores standardizes the data and minimizes the impact of extreme values. Rescaling the transformed scores to a range of 0 to 10 enhances interpretability and facilitates visualization in network analysis.

Toxicogenomics data from the CTD (Database; Davis et al. 2025) was used as an independent data source to help establish the optimal scaled score as cut-off, and to test the safetyome. Disease-associated targets from the CTD were retrieved, and diseases were mapped to MedDRA SOC terms using NLP. Disease SOCs from the CTD were used to compared to phenotypic SOCs from safetyome, for targets at each group of scaled score percentile ranging from 10% to 90%. Here, percentiles denote percentile ranks calculated from the final 0 to 10 scaled score. The disease-phenotype matching increases from 73.2% to 78.0% with the increasing of scaled score from 10% to 50%. The matching then decreases steadily and reaches a plateau after scaled score reaches 70%, i.e., targets with 70%, 80%, 90% scaled scores all showed 70.9% matching between diseases from the CTD and phenotypes from the safetyome, as illustrated in Fig. S3A. With this empirical evidence, a scaled score of 50%, where highest matching diseases in the CTD were observed, is used as cutoff to reliably select proteins with stronger evidence, which resulted in safetyome containing a total number of 11,713 targets. The orthogonal testing using the CTD also provides a systematic framework to rapidly curate off-targets with safety concerns. It is also important to note that CTD may bear shared data sources with the databases used to build the safetyome, hence is used as an orthogonal testing instead of an entirely unbiased validation.

Using the 50% scaled score cutoff, Fig. S3B compares enriched SOC categories with their mapped counterparts, ranked by match percentage. Notably, SOCs such as cardiac, psychiatric, and eye disorders show high match rates exceeding 90%, indicating a strong correlation. In contrast, infectious diseases exhibit match rates below 40%, likely due to the complexities associated with a diverse array of pathogens and host interactions, which might not be adequately reflected by the human genetics and pharmacology evidence utilized in our approach.

### Characterization of safetyome

**Fig. 2.**
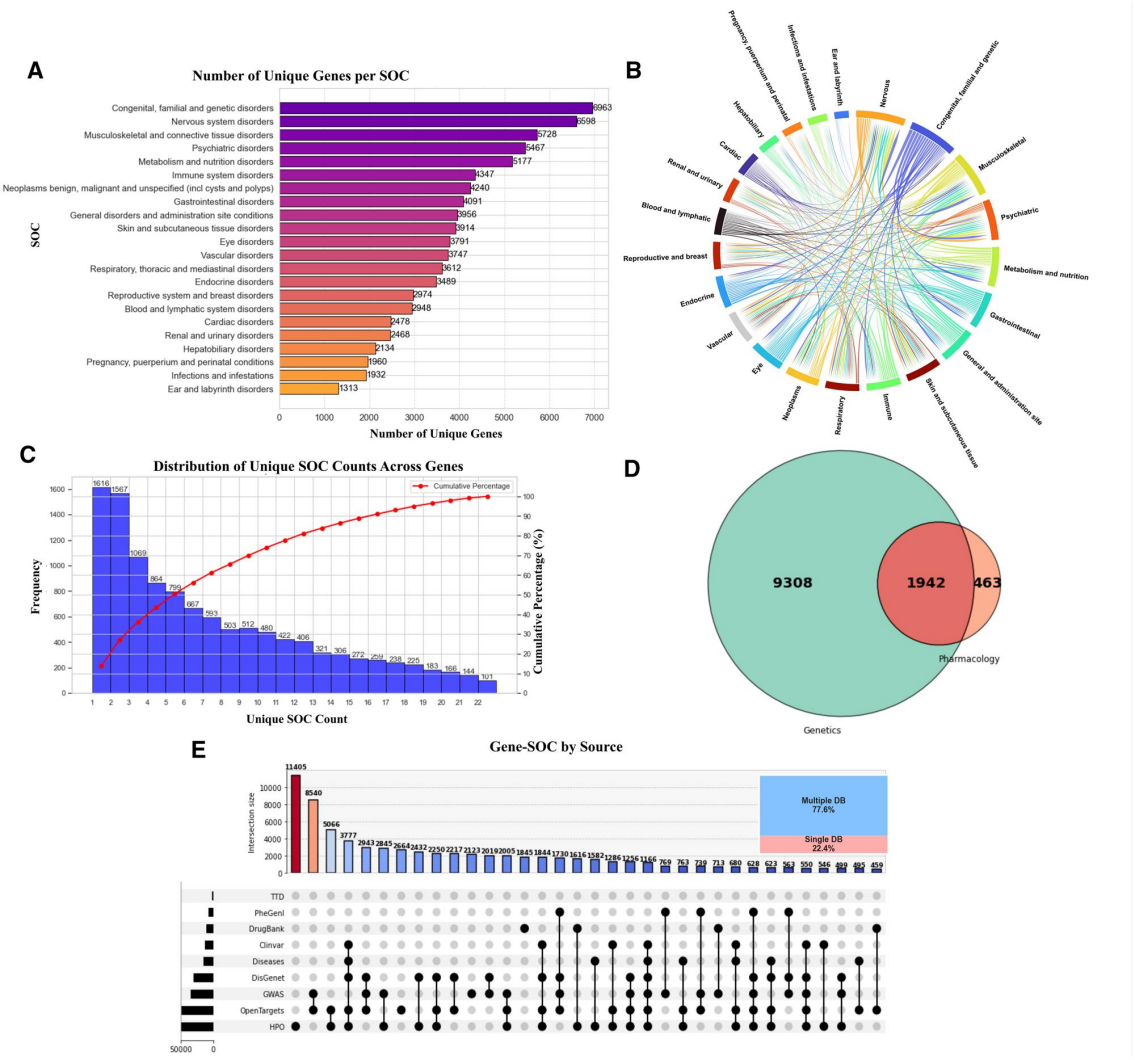
Distribution of unique targets across SOCs within safetyome. A) A bar chart depicting the number of unique genes associated with each SOC. (B) A circular chord diagram illustrating the interconnectivity between genes and their corresponding SOCs. C) Distribution of unique SOC counts across individual genes. The left y-axis indicates frequency, while the right y-axis shows percentage. D) A Venn diagram showing the overlap between genes derived from genetics and those from pharmacological datasets. E) An UpSet plot depicting the distribution of gene-SOC associations by data source. The bar chart in the top left quantifies the number of associations from individual sources, while the intersection matrix below highlights the overlap and unique gene-SOC pairs across databases. The pie chart in the upper right corner indicates the percentage of associations originating from multiple versus single sources.

Fig. 2 illustrates the analysis of the distribution of unique targets across SOCs within the safetyome and highlights the overlap between genetic and pharmacological datasets. Notably, congenital, familial, and genetic disorders are associated with the highest number of targets, underscoring the hereditary nature for this particular SOC. Conversely, categories such as infections, immune-related conditions, and ear disorders exhibit relatively fewer associated targets, which may be attributed to the complex interplay between genetic and environmental factors, as well as the less well-defined pharmacological targets in these areas (Cohen and Phillips 2012; Carey and Palumbos 2016; McInnes and Gravallese 2021; Lüleci and Yılmaz 2022). The circular chord diagram (Fig. 2B) depicts the interconnections among different SOC classes, illustrating that certain targets exert pleiotropic effects on multiple organ systems. For instance, a substantial interconnectivity between the two SOCs terms, psychiatric and nervous system disorders, suggests shared neurobiological mechanisms (Lee et al. 2021). Similarly, hepatobiliary disorders exhibit substantial overlap in targets with metabolic and nutritional disorders, reflecting interconnected physiological pathways (Eslam et al. 2020; Gan et al. 2025). Endocrine disorders also show high overlap with metabolic and nutritional disorders, reinforcing the close interplay between hormonal regulation and metabolic processes (Islam et al. 2024). Additionally, metabolic and nutritional disorders demonstrate the strongest overlap with nervous system disorders, highlighting the intricate relationship between metabolism and neurological functions (Poddar et al. 2021).On the other hand, more than 50% of the targets analyzed demonstrate highly specialized interactions, i.e., associated with ≤ 5 distinct systems (Fig. 2C). This observation indicates that a substantial number of these targets are predominantly engaged with specific organ systems, rather than exhibiting extensive interactions across multiple SOCs.

The value of human genetics as a source of off-target identification is significant. The intersection analysis depicted in Fig. 2D reveals that there are 1,942 targets common to both genetic and pharmacological datasets, 463 targets exclusive to pharmacological contexts and a notable 9,308 targets solely identified from human genetics. Despite the huge and ever-growing knowledge in human genetics and unraveled translational values comparing to other empirical parameters such as hit rates, the off-target selection only began to leverage human genetics in recent years, with only a handful of published examples (Deaton et al. 2019; Liu et al. 2021). Fig. 2E explores the distribution of target-SOC associations by data source, demonstrating that the majority of these associations (77.8%) are validated across multiple databases, which increase the confidence of this iteration of the safetyome.

We subsequently investigated the cellular localization, protein class, and protein functions of targets contained in the safetyome. As depicted in Fig. S4A, the cytoplasm houses the highest number of targets (3,875) that play a crucial role in metabolic processes, protein translation, and intracellular signaling. The membrane-associated category follows with 2,713 targets, comprising proteins involved in cellular signaling, transport, and structural integrity. Many of these proteins are pharmacological targets, such as receptors, ion channels, and adhesion molecules. Additionally, secreted proteins (1,329) constitute a significant category, including hormones, growth factors, cytokines, and extracellular matrix components that are essential for intercellular communication and immune responses. Other subcellular compartments such as mitochondrion, endoplasmic reticulum, Golgi apparatus, lysosome and synapse also show notable target representation in safetyome, supporting essential functions like energy metabolism, protein trafficking, degradation and neurotransmission.

Functionally (Fig. S4B), the safetyome is dominated by metabolite interconversion enzymes (1,394) and protein-modifying enzymes (1,124), which drive core metabolic and regulatory processes. Gene-specific transcription regulators (905), transporters (747), and transmembrane signal receptors (579) are also highly represented in safetyome. Additional functional categories—including intercellular signaling molecules, cell adhesion molecules, immune defense proteins, structural proteins, and calcium-binding proteins—contribute to tissue organization, immune function, and cellular stability. The broad functional distribution highlights the diverse and vital functional roles of the proteins captured in safetyome, ranging from structural components to enzymatic and regulatory molecules.

Furthermore, Fig. S4C illustrates the overlap between targets in the previous SOTP panel by (Liu et al. 2021) and the current safetyome. The SOTP of 2,813 targets encompass only central nervous system, cardiovascular, and respiratory organ systems. The full safetyome described herein comprises a total of 11,713 targets. Notably, 2,699 targets are shared between both panels. Hence, while there is a substantial retention of targets from the previous version, the new panel introduces an additional 9,014 targets, significantly enhancing its overall coverage especially to key safety organs beyond CV, CNS, and respiratory.

To provide a concrete translational anchor for this expansion, we assessed the associated SOCs and scaled scores of CEREP SafetyScreen44 panel targets, a set of well-established off-targets routinely screened in *in vitro* secondary pharmacology across pharmaceutical companies (Bowes et al. 2012). Consistent with their known safety relevance, these 44 targets show significantly higher median scaled scores than non-panel targets (Fig. S5A). We further summarized the top associated SOCs for each SafetyScreen44 target (Fig. S5B), observing SOC patterns that align with recognized liabilities, including prominent enrichment in nervous system/psychiatric and cardiovascular categories alongside additional system-specific signals. For example, canonical neuro/CNS targets (e.g. GRIN1, GABRA1, SLC6A2/3/4, CHRNA4, HTR1A/HTR1A/HTR2A/HTR2B/HTR3A, DRD1/DRD2, MAOA, and CHRM1/2/3) show primary associations with nervous system/psychiatric SOCs, while well-established cardiovascular targets (e.g. SCN5A, KCNH2, KCNQ1, ADRB1/ADRB2, EDNRA, CACNA1C, and PDE3A) link predominantly to cardiovascular SOCs. Consistent with expected biology, ADRB2, CHRM3, and OPRM1 are primarily associated with respiratory disorders, AR with reproductive and endocrine system phenotypes, and NR3C1, HRH1 and LCK with immune-related disorders.

Taken together, the overlap with the prior SOTP panel and the concordance with SafetyScreen44 further demonstrated that the revised safetyome is not only substantially more comprehensive, but also remains robustly grounded to well-validated, industry-relevant safety targets, supporting its practical utility for interpreting *in vitro* findings, understanding liabilities for new off-targets, and prioritizing potential *in vivo* safety risks.

### Target prioritization and core safetyome panel of 500 targets

With its significant size, prioritization of targets included in the safetyome using additional biologically relevant metrics might be necessary (Fig. S6A). To this end, tissue distribution pattern and the conservation across species were used to rank targets. Áine *et al.* has shown tissue specificity can be used to predict side effects of administrated drugs (Duffy et al. 2020). Additionally, highly tissue specific targets have higher association with human diseases in comparison to their pan expressed counterparts (Dvir et al. 2022). Tissue specificity can be quantified using the Tau index (Kryuchkova-Mostacci and Robinson-Rechavi 2017). Target with a Tau index close to 1 is more specifically expressed in one tissue, while target with a Tau index close to 0 is ubiquitously expressed across all tissues studied. Similarly, targets that are conserved across species might be more important in their biological functions (Rancati et al. 2018; Oz et al. 2022). Conservation scores were computed based on the median of two parameters: (i) the identities for gene sequences across species; and (ii) the confidence of gene orthologs identified across species retrieved from ensemble database (Harrison et al. 2024; Project). The Tau index and gene conservation scores were used, in combination with scaled score, to rank targets in safetyome, as detailed in Materials and Methods. The resulting top 500 prioritized targets were included into a core panel. As shown in Fig. S6B and Table S2, the resulting core panel showed a further improvement from the full safetyome, in terms of matching between targets expression and phenotype, validating the benefits provided by further incorporating the biologically relevant information from expression and conservation. We also noticed “Nervous system disorders-Brain” has the highest number of matching SOC-tissue pair targets, which means that phenotypes, tissue specificity and conservation play important roles in the brain. Targets with different prioritizing metrics are available at Supplemental Table 3. The 500 targets included in the core panel are listed in Supplemental Table 4.

### Phenotype based gene (or protein) sets

**Fig. 3.**
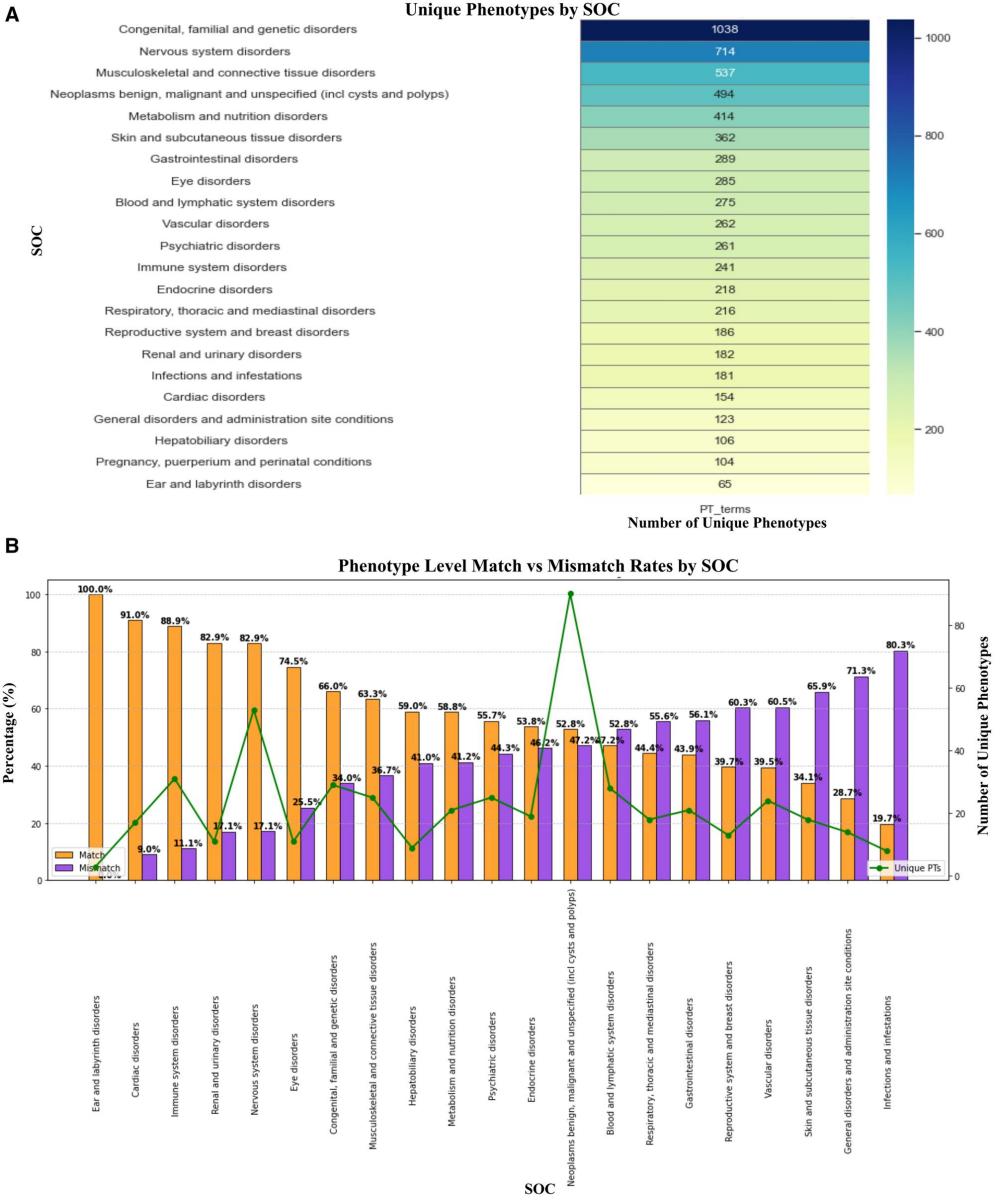
Distribution of unique phenotype across SOCs and validation of phenotype-based gene sets. A) Number of unique phenotype-based gene sets associated with each SOC category. B) Match rates of phenotypes (PT level) enriched through statistical overrepresentation analysis of gene sets in CTD compared to the mapped phenotypes via NLP.

When mapped to preferred term (PT) level, which is closest to the specific phenotype terms encountered during preclinical and clinical developments, targets can be grouped together based on a given phenotypic term. The resulting set can be used as gene or protein set for issue resolution. To gain confidence, we retained gene-PT pairs with evidence present in at least 2 databases. A total of 3,453 unique PT-level phenotypes (panels) were obtained, encompassing 10,892 unique targets. Grouping these panels into organ systems (SOCs), we observed that the systems containing the most panels are: Congenital, familial, and genetic disorders (1,038 panels), followed by nervous system disorders (714 panels) and musculoskeletal disorders (537 panels), as illustrated in Fig. 3A. Whereas the smallest sets come from systems of pregnancy, puerperium, and perinatal conditions (104 panels), as well as ear and labyrinth disorders (65 panels).

To systematically test these phenotypic sets, we performed PT-level enrichment analysis using the CTD, in a fashion similar to the analysis on the SOC level. Diseases from the CTD were first mapped directly to MedDRA PT terms using NLP, resulting in NLP-mapped PT terms. Subsequently, enrichment analysis was performed on our phenotypic panels, yielding enriched PT-level terms. The accuracy of phenotype mapping was evaluated by calculating the exact match rate between the NLP-mapped PTs and the enriched PTs. The average PT-level match rate was 55.5% (Fig. S7), which shows great promises considering totally unbiased real-world data used for testing, as well as the largest scale in a cohesive fashion to our knowledge and attempt to compose and validate the 3,000+ specialized panels. The average matching rate reflects the mixture of groups with high and low matchings, as detailed below.

Examining individual panels further revealed variation in match rates (Fig. 3B). Phenotypes in five systems (SOCs) demonstrated notably high PT-level match rates exceeding 80%, which are “Ear and labyrinth disorders” (100%), “Cardiac disorders” (91.0%), “Immune system disorders” (88.9%), “Renal and urinary disorders” (82.9%), and “Nervous system disorders” (82.8%). Phenotypes in “Eye disorders” also demonstrated a relatively high match rate of 74.5%, though with notable mismatches for several enriched PT terms. Closer evaluation revealed that these mismatches mostly arose from minor semantic variations rather than substantial biological discrepancies. For example, while the NLP-mapped CTD phenotype “macular degeneration” closely matched the enriched phenotype “age-related macular degeneration”, these terms represent closely related conditions. Similarly, NLP-mapped “congenital night blindness” and enriched “night blindness” are conceptually similar, differing primarily by specificity. Additionally, “Sjogren’s syndrome” (mapped PT) and “systemic lupus erythematosus”(enriched PT) represent different but biologically and clinically related autoimmune disorders, explaining their co-occurrence. Thus, most observed mismatches in this category do not indicate substantial errors but rather reflect subtle differences in phenotypic granularity or disease classifications. In contrast, phenotypes in two systems yielded the lowest matching, which are “Infections and infestations”(19.6%), and “General disorders and administration site conditions” (28.7%). These lower concordances likely result from greater heterogeneity in phenotype descriptions and inherent molecular complexities, which complicate accurate definition and identification of associated or causal genes.

### PhenoSOC and PhenoPT app

A key outcome of this work is the development of a user-friendly PhenoSOC and PhenoPT APP (https://jnjtst.shinyapps.io/safetyome_v1-1/). PhenoSOC is a resource to instantaneously and systematically annotate for a query target, to homogenize SOC terms. In PhenoSOC, human target of interest can be queried using the symbol from the HUGO gene nomenclature committee (HGNC). Upon clicking “submit,” the SOCs, the supporting databases, as well as the scaled score will be displayed in both schematic and table formats. The table can also be downloaded for further mining. PhenoPT provides the list of (likely causal) targets for a given phenotype of interest at the PT level, which were pre-populated in the query box. The output will be a list of targets that are implicated in the phenotype of interest based on genetics and/or pharmacology evidence. The supporting databases and scaled scores are also provided. The table can easily be downloaded into excel and sorted based on the strength of evidence (scaled scores). In summary, PhenoSOC approaches the query from the perspective of the target, whereas PhenoPT approaches the query from the phenotype perspective.

## Discussion

Selecting proteins to go into an off-target panel is historically a manual process, with careful considerations from biology, legacy experiences, hit rates, and assay availability etc. Although valuable in many aspects, these considerations can be highly variable, hard to scale up to proteome or genome wide levels and often circling back to the existing off-targets instead of identifying new ones. Most importantly, despite their well-established biological function, binding to or functionally disrupting these off-target proteins may lead to minimal to no adverse phenotypes, due to complex biology, redundant and compensatory pathways and so on (Nowak et al. 1997; Gibson and Spring 1998; Gu et al. 2003; Vavouri et al. 2008; Laruson et al. 2020). These traditional approaches to identify off-target proteins also do not adequately and systematically address translatability. With the goal of bringing translatability into target-based safety assessment, we developed a comprehensive and scalable approach that systematically integrates human genetic and pharmacological data to define safety-relevant off-target proteins and phenotypic panels. There are three key advancements of this study in comparison to the previous pilot work (Deaton et al. 2019; Liu et al. 2021). One is the expansion of the previously defined SOTP (Liu et al. 2021), which was limited to 5 systems, into a comprehensive resource spanning 22 systems and including key systems relevant to safety such as hepatic, renal, endocrine, and dermatologic systems. The second is the systematically generated panels covering over 3,000 phenotypes annotated by genetic and pharmacological data. Each phenotype panel is composed of targets whose genotypical or pharmacological disturbances are known to have phenotypical manifestations, providing additional relevance. Lastly, both the safetyome full and core panels as well as the 3,000 specialized panels were successfully tested using a large dataset from an orthogonal source (CTD). The full and core panels are best suited for general screen during early preclinical development, while the specialized panels could be of value for issue resolution when a particular phenotype is being investigated. The important off-targets identified from the general panels (core or full) might also inform subsequent *in vivo* studies, i.e., targets linking to bleeding risk might prompt additional end point such as coagulation assays.

The integration of human genetics in safety assessments is increasingly recognized as a transformative advance and could address the gap of informing human *in vitro* to *in vivo* translatability systematically. Prior studies, noticeably by Nelson et al., Nguyen et al., and Carss et al., consistently demonstrated that drug targets with genetic associations to phenotypically similar traits are significantly more likely to manifest on-target and off-target clinical effects (Nelson et al. 2015; Nguyen et al. 2019; Carss et al. 2023). A recent meta-analysis by Minikel and Nelson further revealed that drugs whose targets are genetically linked to similar traits are over twofold more likely to cause the corresponding side effects—an enrichment that persists even after controlling for confounding indication effects (Minikel and Nelson 2025). Moreover, structural biology provided additional insights. Schaafsma and Vihinen demonstrated that disease-associated proteins, particularly those implicated in cancer, harbor a significantly higher proportion of deleterious amino acid substitutions compared to non-disease proteins, indicating heightened vulnerability to functional disruption (Schaafsma and Vihinen 2017). These findings underscore the unraveled translational value of anchoring safety assessments in human genetic evidence, which was only recently incorporated in off-target selection, together with indication-based pharmacology, to help with the challenging task of establishing causal relationship between target and phenotypic outcome (Deaton et al. 2019; Liu et al. 2021). Another possible application comes from the reverse side, i.e., to round up (off)-targets that are phenotypically “benign,” based on the assumption that targets lacking genetic and pharmacological associations may represent lower-priority safety concerns under current knowledge. Such “benign” off-target list may be of value during drug development, particularly with genome- or proteome-wide screenings. However, we did not include the iteration of such “benign” list in the present work as there may be “false negatives” that result from current gaps in knowledge/evidence. With the steady and rapidly expanding knowledge from human genetics, if abundant evidence can be obtained to unambiguously determine the lack of mutations, or lack of phenotypic consequence of mutations, this idea may be realized.

To improve phenotype standardization and interpretability across various sources, we developed a multi-layered NLP pipeline that substantially expanded mapping coverage. This methodological advancement overcomes a long-standing challenge posed by the inconsistent and ambiguous representation of phenotypic descriptors in genetic and pharmacological data, which has hindered cross-database integration and the reliability of gene-to-phenotype associations (Dattani et al. 2022; Lin et al. 2023). The integration of additional prioritization metrics, including tissue specificity and evolutionary conservation across species, constitutes another major methodological refinement. These quantifiable metrics were chosen for their established links to functional importance and translational relevance: Genes with high tissue specificity are more likely to mediate organ-selective toxicities, while conserved genes often encode essential biological functions with higher phenotypic impact upon perturbation. Prioritizing targets based on these parameters enables the identification of those most likely to mediate phenotypically consequential outcomes, thus supporting more efficient resource allocation in safety screening. Another crucial contribution is the establishment of over 3000 phenotype-specific gene sets aligned to MedDRA Preferred Terms, providing a high-resolution, ontology-linked resource for mechanistic investigations of adverse events and facilitate issue resolution when unexpected phenotypes emerge in preclinical or clinical development. To ensure accessibility and real-world usability, our interactive web-based tool enables intuitive query, visualization, and download of both the safetyome and specialized panels, offering a scalable, evidence-based, and biologically grounded platform for early risk identification, target prioritization, and mechanistically informed safety screening in the era of precision therapeutics.

Despite the strengths and broad utility of this framework, several limitations should be acknowledged. First, our analyses rely on publicly available databases and literature-curated sources and, while extensive, are not free from bias or gaps. Genetic association data can be skewed towards well-studied diseases and common variants, whereas some phenotypes relevant to drug safety (especially rare or idiosyncratic phenotypes) may lack comprehensive genetic supports. We mitigated these issues by cross-referencing multiple evidence streams, but the possibility of missing gene–phenotype links remains. Secondly, phenotype mapping to MedDRA terms, while essential for regulatory alignment on safety, introduced semantic compression as it may not fully capture the nuance of complex or composite traits observed in GWAS and rare disease datasets. For example, traits (e.g. “hip circumference adjusted for BMI,” “waist-to-hip ratio adjusted for BMI” or “long face”) cannot be faithfully translated into standardized adverse event terminology. Mapping to additional vocabulary may further help improve the coverage especially at organ system level. Third, we did not explicitly incorporate pharmacokinetic (PK) and exposure data of pharmacology evidence into our safety annotation. Our framework flags potential intrinsic liabilities of targeting a protein, but whether a particular drug will manifest that liability also depends on drug-specific properties such as tissue distribution, metabolism, and dosage. For example, a target might be tied to pulmonary toxicity in our data, however, the risk might be negligible if the new therapy cannot penetrate the lung or has very low lung tissue distribution. The absence of PK context means our panels are leaning more towards sensitivity (capturing any plausible risk) over specificity. Lastly, there are also caveats introduced from the quantitative metrices used in our study, for example, the likelihood of “data leakage or overestimation” by citing the same source across databases, and the Tau index used for expression. When citing same data source across multiple databases, the scaled scores may be inflated as they were derived from the total number of occurrences of a target in all 9 databases. This potential redundancy issue from citing the same source may also exist in CTD. For the Tau index, the basal expression and the differences in affinity across species may vary. Where our current practice remain valid first attemp to suit the scaling up, incorporating these considerations in the future, with expanding *in vitro* data, may further increase the accuracy of prioritization. Despite these limitations, we have strived to maintain a careful balance in interpretation and flagging the potential risks indicated by the data, while acknowledging that real-world drug safety is multi-factorial. Importantly, these limitations do not detract from the overall contribution of this work; instead, they underscore opportunities for future refinement and the importance of continuously incorporating emerging data as the field advances. For example, one future direction could be incorporating PK estimations to better prioritize target-based risk assessment. The wet-lab testing, likely using proteomics and transcriptomics given the size of targets, i.e., 50 to hundreds for each phenotype, would also be valuable, although collecting human samples treated with sizable number of phenotype-inducing drugs will remain a big hurdle. Another valuable addition to the computational pipeline will be to incorporate the directionalities of regulation of a given target on the phenotype of interest, extracted from genetics and pharmacology evidence.

In conclusion, this study provides a systematic and translational framework for anticipating drug safety liabilities by uniting human genetic and pharmacological evidence at an unprecedented scale. Our findings reinforce the principle that “lessons from nature”, as reflected in human genetic variation and real-world pharmacological observations, can be harnessed to better understand and mitigate unintended consequences of therapeutic intervention. Enabled by a user-friendly interface, we anticipate that the readily adoption of this framework for both prospective screening and retrospective issue resolution.

## Supplementary Material

kfag021_Supplementary_Data

## References

[kfag021-B2] Bodenreider O. 2004. The unified medical language system (umls): integrating biomedical terminology. Nucleic Acids Res. 32:D267–270. 14681409 10.1093/nar/gkh061 PMC308795

[kfag021-B3] Bonferroni C. 1936. Teoria statistica delle classi e calcolo delle probabilita. Pubblicazioni Del R Istituto Superiore Di Scienze Economiche e Commericiali Di Firenze. 8:3–62.

[kfag021-B4] Bowes J , Brown AJ, Hamon J, Jarolimek W, Sridhar A, Waldron G, Whitebread S. 2012. Reducing safety-related drug attrition: the use of in vitro pharmacological profiling. Nat Rev Drug Discov. 11:909–922. 23197038 10.1038/nrd3845

[kfag021-B5] Brennan RJ , Jenkinson S, Brown A, Delaunois A, Dumotier B, Pannirselvam M, Rao M, Ribeiro LR, Schmidt F, Sibony A, et al 2024. The state of the art in secondary pharmacology and its impact on the safety of new medicines. Nat Rev Drug Discov. 23:525–545. 38773351 10.1038/s41573-024-00942-3

[kfag021-B6] Buniello A , Suveges D, Cruz-Castillo C, Llinares MB, Cornu H, Lopez I, Tsukanov K, Roldan-Romero JM, Mehta C, Fumis L, et al 2025. Open targets platform: facilitating therapeutic hypotheses building in drug discovery. Nucleic Acids Res. 53:D1467–D1475. 39657122 10.1093/nar/gkae1128 PMC11701534

[kfag021-B7] Carey JC , Palumbos JC. 2016. Advances in the understanding of the genetic causes of hearing loss in children inform a rational approach to evaluation. Indian J Pediatr. 83:1150–1156. 26743077 10.1007/s12098-015-1941-x

[kfag021-B8] Carss KJ , Deaton AM, Del Rio-Espinola A, Diogo D, Fielden M, Kulkarni DA, Moggs J, Newham P, Nelson MR, Sistare FD, et al 2023. Using human genetics to improve safety assessment of therapeutics. Nat Rev Drug Discov. 22:145–162. 36261593 10.1038/s41573-022-00561-w

[kfag021-B9] Cerezo M , Sollis E, Ji Y, Lewis E, Abid A, Bircan KO, Hall P, Hayhurst J, John S, Mosaku A, et al 2025. The nhgri-ebi gwas catalog: standards for reusability, sustainability and diversity. Nucleic Acids Res. 53:D998–D1005. 39530240 10.1093/nar/gkae1070 PMC11701593

[kfag021-B10] Clinvar. [accessed 2024 July]. https://www.clinicalgenome.org/data-sharing/clinvar/

[kfag021-B11] Cohen M , Phillips JA. 3rd. 2012. Genetic approach to evaluation of hearing loss. Otolaryngol Clin North Am. 45:25–39. 22115680 10.1016/j.otc.2011.08.015

[kfag021-B12] Comparative toxicogenomics database. [accessed 2025 March]. https://ctdbase.org/reports/CTD_genes_diseases.csv.gz

[kfag021-B13] Dattani S , Howard DM, Lewis CM, Sham PC. 2022. Clarifying the causes of consistent and inconsistent findings in genetics. Genet Epidemiol. 46:372–389. 35652173 10.1002/gepi.22459 PMC9544854

[kfag021-B14] Davis AP , Wiegers TC, Sciaky D, Barkalow F, Strong M, Wyatt B, Wiegers J, McMorran R, Abrar S, Mattingly CJ. 2025. Comparative toxicogenomics database’s 20th anniversary: update 2025. Nucleic Acids Res. 53:D1328–D1334. 39385618 10.1093/nar/gkae883 PMC11701581

[kfag021-B15] Deaton AM , Fan F, Zhang W, Nguyen PA, Ward LD, Nioi P. 2019. Rationalizing secondary pharmacology screening using human genetic and pharmacological evidence. Toxicol Sci. 167:593–603. 30346593 10.1093/toxsci/kfy265 PMC6358245

[kfag021-B16] Disease-gene associations mined from literature database [accessed 2024 July]. https://diseases.jensenlab.org/Downloads

[kfag021-B17] Drugbank 5.1.12. [accessed 2024 October]. https://www.drugbank.com/.

[kfag021-B18] Duffy Á , Verbanck M, Dobbyn A, Won HH, Rein JL, Forrest IS, Nadkarni G, Rocheleau G, Do R. 2020. Tissue-specific genetic features inform prediction of drug side effects in clinical trials. Sci Adv. 6:1–11. 10.1126/sciadv.abb6242 PMC11206454 32917698

[kfag021-B19] Dvir E , Shohat S, Flint J, Shifman S. 2022. Identification of genetic mechanisms for tissue-specific genetic effects based on crispr screens. Genetics. 222:1–11. 10.1093/genetics/iyac134 PMC9630981 36063051

[kfag021-B20] Eslam M , Newsome PN, Sarin SK, Anstee QM, Targher G, Romero-Gomez M, Zelber-Sagi S, Wai-Sun Wong V, Dufour J-F, Schattenberg JM, et al 2020. A new definition for metabolic dysfunction-associated fatty liver disease: an international expert consensus statement. J Hepatol. 73:202–209. 32278004 10.1016/j.jhep.2020.03.039

[kfag021-B21] Fey MF , O’Reilly S, Awada AH, Crowley J, Gelmon KA. 2024. Diligent use of meddra terminology and preferred term selection in safety reports of clinical trials. Curr Opin Oncol. 36:418–420. 39106404 10.1097/CCO.0000000000001056

[kfag021-B22] Gan C , Yuan Y, Shen H, Gao J, Kong X, Che Z, Guo Y, Wang H, Dong E, Xiao J. 2025. Liver diseases: epidemiology, causes, trends and predictions. Signal Transduct Target Ther. 10:33. 39904973 10.1038/s41392-024-02072-z PMC11794951

[kfag021-B23] Gargano MA , Matentzoglu N, Coleman B, Addo-Lartey EB, Anagnostopoulos AV, Anderton J, Avillach P, Bagley AM, Bakstein E, Balhoff JP, et al 2024. The human phenotype ontology in 2024: phenotypes around the world. Nucleic Acids Res. 52:D1333–D1346. 37953324 10.1093/nar/gkad1005 PMC10767975

[kfag021-B24] Gibson TJ , Spring J. 1998. Genetic redundancy in vertebrates: polyploidy and persistence of genes encoding multidomain proteins. Trends Genet. 14:46–49. discussion 49–50. 9520595 10.1016/s0168-9525(97)01367-x

[kfag021-B25] Grissa D , Junge A, Oprea TI, Jensen LJ. 2022. Diseases 2.0: A weekly updated database of disease-gene associations from text mining and data integration. Database (Oxford). 2022. 10.1093/database/baac019 PMC9216524 35348648

[kfag021-B26] Gu Y , Tinn R, Cheng H, Lucas M, Usuyama N, Liu X, Naumann T, Gao J, Poon H. 2022. Domain-specific language model pretraining for biomedical natural language processing. ACM Trans Comput Healthc. 3:1–23.

[kfag021-B27] Gu Z , Steinmetz LM, Gu X, Scharfe C, Davis RW, Li WH. 2003. Role of duplicate genes in genetic robustness against null mutations. Nature. 421:63–66. 12511954 10.1038/nature01198

[kfag021-B28] Harrison PW , Amode MR, Austine-Orimoloye O, Azov AG, Barba M, Barnes I, Becker A, Bennett R, Berry A, Bhai J, et al 2024. Ensembl 2024. Nucleic Acids Res. 52:D891–D899. 37953337 10.1093/nar/gkad1049 PMC10767893

[kfag021-B29] Henrie A , Hemphill SE, Ruiz-Schultz N, Cushman B, DiStefano MT, Azzariti D, Harrison SM, Rehm HL, Eilbeck K. 2018. Clinvar miner: demonstrating utility of a web-based tool for viewing and filtering clinvar data. Hum Mutat. 39:1051–1060. 29790234 10.1002/humu.23555 PMC6043391

[kfag021-B30] Human phenotype ontology. [accessed 2024 July]. https://hpo.jax.org/

[kfag021-B31] International HapMap C. 2005. A haplotype map of the human genome. Nature. 437:1299–1320. 16255080 10.1038/nature04226 PMC1880871

[kfag021-B32] Islam MS , Wei P, Suzauddula M, Nime I, Feroz F, Acharjee M, Pan F. 2024. The interplay of factors in metabolic syndrome: understanding its roots and complexity. Mol Med. 30:279. 39731011 10.1186/s10020-024-01019-y PMC11673706

[kfag021-B33] Jenkinson S , Schmidt F, Rosenbrier Ribeiro L, Delaunois A, Valentin JP. 2020. A practical guide to secondary pharmacology in drug discovery. J Pharmacol Toxicol Methods. 105:106869. 32302774 10.1016/j.vascn.2020.106869

[kfag021-B34] Kang Y , Sun Z, Wang S, Huang Z, Wu Z, Ma X. 2021. Metamap: Supporting visual metaphor ideation through multi-dimensional example-based exploration. Paper presented at: Proceedings of the 2021 CHI conference on human factors in computing systems. Yokohama, Japan: Association for Computing Machinery.

[kfag021-B35] Knox C , Wilson M, Klinger CM, Franklin M, Oler E, Wilson A, Pon A, Cox J, Chin NEL, Strawbridge SA, et al 2024. Drugbank 6.0: the drugbank knowledgebase for 2024. Nucleic Acids Res. 52:D1265–D1275. 37953279 10.1093/nar/gkad976 PMC10767804

[kfag021-B36] Kryuchkova-Mostacci N , Robinson-Rechavi M. 2017. A benchmark of gene expression tissue-specificity metrics. Brief Bioinform. 18:205–214. 26891983 10.1093/bib/bbw008 PMC5444245

[kfag021-B37] Landrum MJ , Chitipiralla S, Brown GR, Chen C, Gu B, Hart J, Hoffman D, Jang W, Kaur K, Liu C, et al 2020. Clinvar: improvements to accessing data. Nucleic Acids Res. 48:D835–D844. 31777943 10.1093/nar/gkz972 PMC6943040

[kfag021-B38] Laruson AJ , Yeaman S, Lotterhos KE. 2020. The importance of genetic redundancy in evolution. Trends Ecol Evol. 35:809–822. 32439075 10.1016/j.tree.2020.04.009

[kfag021-B39] Lee PH , Feng YA, Smoller JW. 2021. Pleiotropy and cross-disorder genetics among psychiatric disorders. Biol Psychiatry. 89:20–31. 33131714 10.1016/j.biopsych.2020.09.026 PMC7898275

[kfag021-B40] Lin L , Pan H, Qi Y, Ma Y, Qiu L. 2023. Reasons and resolutions for inconsistent variant interpretation. Hum Mutat. 2023:4955235. 40225163 10.1155/2023/4955235 PMC11919222

[kfag021-B41] Liu X , Zhang Y, Ward LD, Yan Q, Bohnuud T, Hernandez R, Lao S, Yuan J, Fan F. 2021. A proteomic platform to identify off-target proteins associated with therapeutic modalities that induce protein degradation or gene silencing. Sci Rep. 11:15856. 34349202 10.1038/s41598-021-95354-3 PMC8338952

[kfag021-B42] Lonsdale J , Thomas J, Salvatore M, Phillips R, Lo E, Shad S, Hasz R, Walters G, Garcia F, Young N, et al 2013. The genotype-tissue expression (gtex) project. Nat Genet. 45:580–585. 23715323 10.1038/ng.2653 PMC4010069

[kfag021-B43] Lüleci HB , Yılmaz A. 2022. Robust and rigorous identification of tissue-specific genes by statistically extending tau score. BioData Min. 15:31. 36494766 10.1186/s13040-022-00315-9 PMC9733102

[kfag021-B44] McInnes IB , Gravallese EM. 2021. Immune-mediated inflammatory disease therapeutics: past, present and future. Nat Rev Immunol. 21:680–686. 34518662 10.1038/s41577-021-00603-1 PMC8436867

[kfag021-B45] Minikel EV , Nelson MR. 2025. Human genetic evidence enriched for side effects of approved drugs. PLoS Genet. 21: E 1011638. 10.1371/journal.pgen.1011638 PMC11977994 40163513

[kfag021-B46] Medical dictionary for regulatory activities. [accessed 2024 July]. https://www.meddra.org/

[kfag021-B47] Nelson MR , Tipney H, Painter JL, Shen J, Nicoletti P, Shen Y, Floratos A, Sham PC, Li MJ, Wang J, et al 2015. The support of human genetic evidence for approved drug indications. Nat Genet. 47:856–860. 26121088 10.1038/ng.3314

[kfag021-B48] Nguyen PA , Born DA, Deaton AM, Nioi P, Ward LD. 2019. Phenotypes associated with genes encoding drug targets are predictive of clinical trial side effects. Nat Commun. 10:1579. 30952858 10.1038/s41467-019-09407-3 PMC6450952

[kfag021-B49] Nowak MA , Boerlijst MC, Cooke J, Smith JM. 1997. Evolution of genetic redundancy. Nature. 388:167–171. 9217155 10.1038/40618

[kfag021-B50] Open targets. 24.03. [accessed 2024 October]. https://www.opentargets.org/

[kfag021-B51] Oz N , Vayndorf EM, Tsuchiya M, McLean S, Turcios-Hernandez L, Pitt JN, Blue BW, Muir M, Kiflezghi MG, Tyshkovskiy A, et al 2022. Evidence that conserved essential genes are enriched for pro-longevity factors. Geroscience. 44:1995–2006. 35695982 10.1007/s11357-022-00604-5 PMC9616985

[kfag021-B52] Palmer D , Fabris F, Doherty A, Freitas AA, de Magalhães JP. 2021. Ageing transcriptome meta-analysis reveals similarities and differences between key mammalian tissues. Aging (Albany NY). 13:3313–3341. 33611312 10.18632/aging.202648 PMC7906136

[kfag021-B53] Papoian T , Chiu HJ, Elayan I, Jagadeesh G, Khan I, Laniyonu AA, Li CX, Saulnier M, Simpson N, Yang B. 2015. Secondary pharmacology data to assess potential off-target activity of new drugs: a regulatory perspective. Nat Rev Drug Discov. 14:294. 10.1038/nrd3845-c1 25792260

[kfag021-B54] Papoian T , Jagadeesh G, Saulnier M, Simpson N, Ravindran A, Yang B, Laniyonu AA, Khan I, Szarfman A. 2017. Regulatory forum review: utility of in vitro secondary pharmacology data to assess risk of drug-induced valvular heart disease in humans: regulatory considerations. Toxicol Pathol. 45:381–388. 28421966 10.1177/0192623317690609

[kfag021-B55] Pe’er I , Yelensky R, Altshuler D, Daly MJ. 2008. Estimation of the multiple testing burden for genomewide association studies of nearly all common variants. Genet Epidemiol. 32:381–385. 18348202 10.1002/gepi.20303

[kfag021-B56] Pinero J , Ramirez-Anguita JM, Sauch-Pitarch J, Ronzano F, Centeno E, Sanz F, Furlong LI. 2020. The disgenet knowledge platform for disease genomics: 2019 update. Nucleic Acids Res. 48:D845–D855. 31680165 10.1093/nar/gkz1021 PMC7145631

[kfag021-B57] Phenotype-genotype integrator [accessed 2024 July]. https://www.ncbi.nlm.nih.gov/gap/phegeni

[kfag021-B58] Poddar MK , Banerjee S, Chakraborty A, Dutta D. 2021. Metabolic disorder in Alzheimer’s disease. Metab Brain Dis. 36:781–813. 33638805 10.1007/s11011-021-00673-z

[kfag021-B59] Pradeep S. 2024. Investigating word embedding techniques for extracting disease, gene, and chemical relationships from biomedical texts. [accessed 2024 July]. https://dalspace.library.dal.ca/items/7ccdb371-38e5-4159-951e-893aa222b597

[kfag021-B61] Ramos EM , Hoffman D, Junkins HA, Maglott D, Phan L, Sherry ST, Feolo M, Hindorff LA. 2014. Phenotype-genotype integrator (phegeni): synthesizing genome-wide association study (gwas) data with existing genomic resources. Eur J Hum Genet. 22:144–147. 23695286 10.1038/ejhg.2013.96 PMC3865418

[kfag021-B62] Rancati G , Moffat J, Typas A, Pavelka N. 2018. Emerging and evolving concepts in gene essentiality. Nat Rev Genet. 19:34–49. 29033457 10.1038/nrg.2017.74

[kfag021-B60] Rios. 2022. [accessed 2024 July]. https://github.com/AnthonyMRios/rios

[kfag021-B63] Schaafsma GC , Vihinen M. 2017. Large differences in proportions of harmful and benign amino acid substitutions between proteins and diseases. Hum Mutat. 38:839–848. 28444810 10.1002/humu.23236

[kfag021-B64] Scispacy: Fast and robust models for biomedical natural language processing. 2019; Florence, Italy: Association for Computational Linguistics.

[kfag021-B65] The ensembl project. [accessed 2025 January 30]. https://useast.ensembl.org/info/genome/compara/Ortholog_qc_manual.html

[kfag021-B66] The nhgri-ebi catalog of human genome-wide association studies. 1.0. [accessed 2024 July]. https://www.ebi.ac.uk/gwas/docs/file-downloads

[kfag021-B67] Therapeutic target database [accessed 2024 July]. https://idrblab.net/ttd/

[kfag021-B68] Vavouri T , Semple JI, Lehner B. 2008. Widespread conservation of genetic redundancy during a billion years of eukaryotic evolution. Trends Genet. 24:485–488. 18786741 10.1016/j.tig.2008.08.005

[kfag021-B69] Verbist BM , Verheyen GR, Vervoort L, Crabbe M, Beerens D, Bosmans C, Jaensch S, Osselaer S, Talloen W, Van den Wyngaert I, et al, QSTAR Consortium 2015. Integrating high-dimensional transcriptomics and image analysis tools into early safety screening: proof of concept for a new early drug development strategy. Chem Res Toxicol. 28:1914–1925. 26313431 10.1021/acs.chemrestox.5b00103

[kfag021-B70] Whitebread S , Dumotier B, Armstrong D, Fekete A, Chen S, Hartmann A, Muller PY, Urban L. 2016. Secondary pharmacology: screening and interpretation of off-target activities—focus on translation. Drug Discov Today. 21:1232–1242. 27140035 10.1016/j.drudis.2016.04.021

[kfag021-B71] Wishart DS , Feunang YD, Guo AC, Lo EJ, Marcu A, Grant JR, Sajed T, Johnson D, Li C, Sayeeda Z, et al 2018. Drugbank 5.0: a major update to the drugbank database for 2018. Nucleic Acids Res. 46:D1074–D1082. 29126136 10.1093/nar/gkx1037 PMC5753335

[kfag021-B72] Wolf T , Debut L, Sanh V, Chaumond J, Delangue C, Moi A, Cistac P, Rault T, Louf R, Funtowicz M. 2019. Huggingface’s transformers: State-of-the-art natural language processing. arXiv preprint arXiv: 191003771., preprint: not peer reviewed.

[kfag021-B73] Wu T , Hu E, Xu S, Chen M, Guo P, Dai Z, Feng T, Zhou L, Tang W, Zhan L, et al 2021. Clusterprofiler 4.0: a universal enrichment tool for interpreting omics data. Innovation (Camb). 2:100141. 34557778 10.1016/j.xinn.2021.100141 PMC8454663

[kfag021-B74] Yu G , Wang LG, Han Y, He QY. 2012. Clusterprofiler: an r package for comparing biological themes among gene clusters. OMICS. 16:284–287. 22455463 10.1089/omi.2011.0118 PMC3339379

[kfag021-B75] Zhang X , Xu J, Xiao WX. 2013. A new method for the discovery of essential proteins. PLoS One. 8:E58763. 23555595 10.1371/journal.pone.0058763 PMC3605424

[kfag021-B76] Zhou Y , Zhang Y, Zhao D, Yu X, Shen X, Zhou Y, Wang S, Qiu Y, Chen Y, Zhu F. 2024. Ttd: Therapeutic target database describing target druggability information. Nucleic Acids Res. 52:D1465–D1477. 37713619 10.1093/nar/gkad751 PMC10767903

