# Supplementary material for: Safetyome and specialized panels for over 3,000 phenotypes: a systematic and translational approach using human genetics and pharmacology
Source: Toxicol Sci. 2026 Feb 19;209(3):kfag021. doi: 10.1093/toxsci/kfag021 (PMC13016935; doi:10.1093/toxsci/kfag021)
Supplement: kfag021_Supplementary_Data [file kfag021_supplementary_data.zip › 26-Feb-2026_041402_Supplemental_Figures_and_Table_Legends.docx]

**Supplementary Tables Titles**

**Supplementary Table 1.** Summary of SOCs used in safetyome database

**Supplementary Table 2.** Prioritization result of safetyome gene sets. Top 50% performing genes based on scaled score were included for additional tissue specificity tau and conservation score. Targets with unmatched names were removed

**Supplementary Table 3.** Matching results with different prioritization metrics. Different tab indicates different core targets (top 500) selected by different metrics. Var1 indicates organ systems with highest expression and SOC indicates organ system with highest scaled score of selected gene. Matched terms were labeled and computed.

**Supplementary Table 4**. Top 500 targets identified as core safetyome panel.


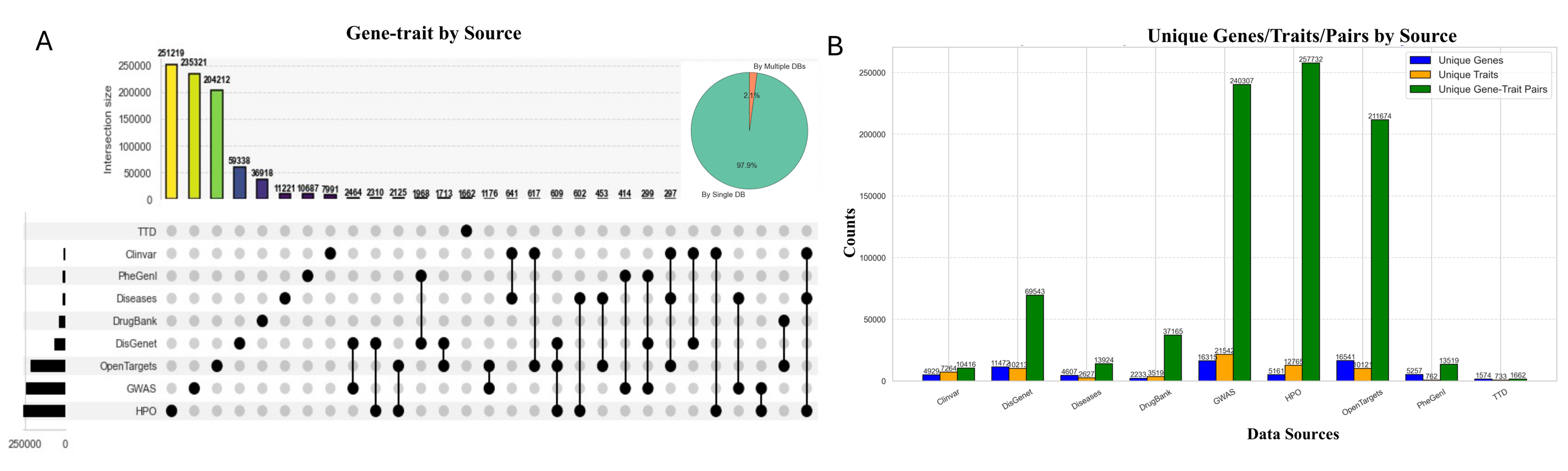


Supplementary Figure 1. Gene-trait and unique gene distribution by data sources. (A) Gene-trait associations by source. (B) Distribution of unique genes/traits/pairs by source.


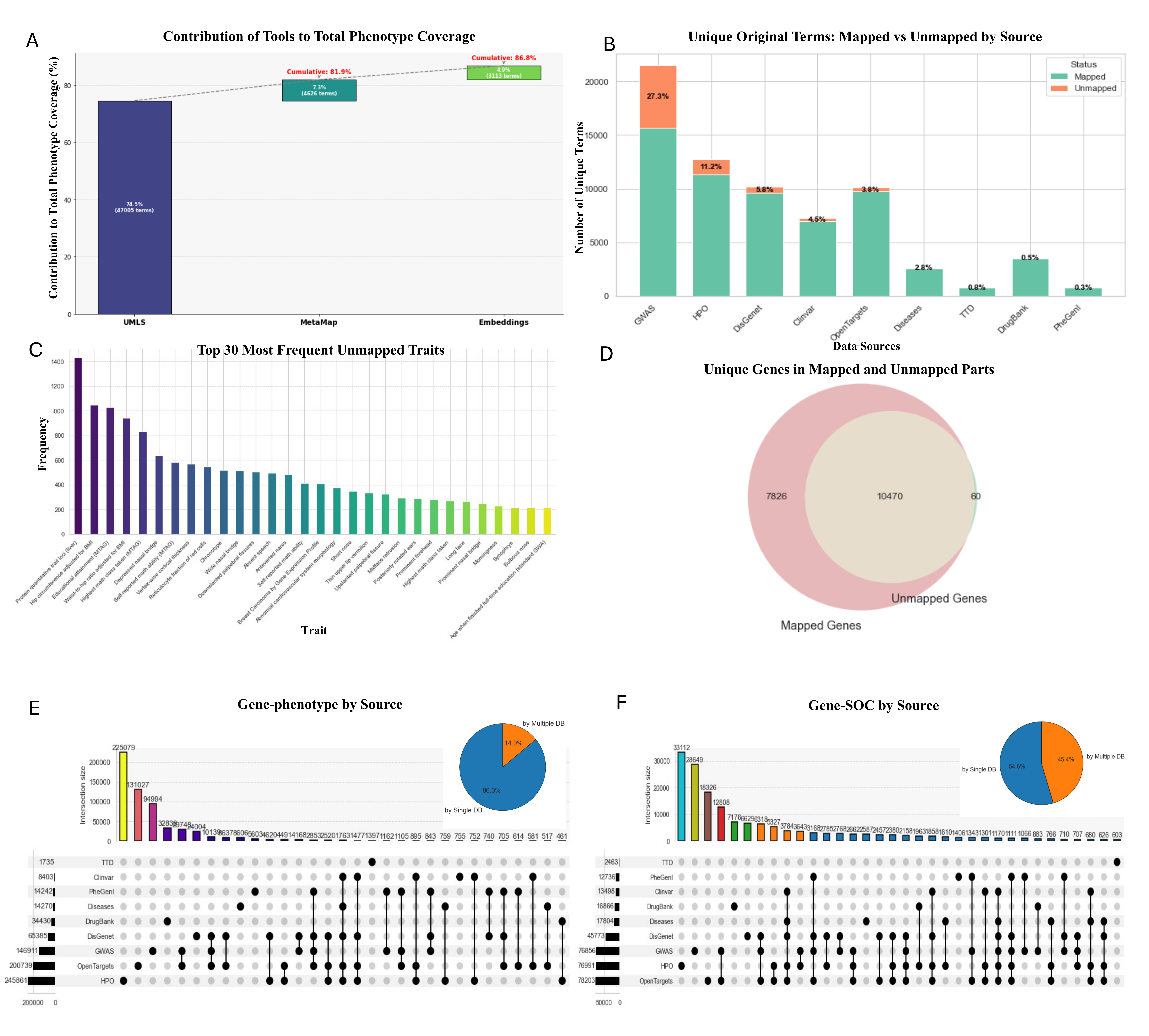
Supplementary Figure 2. Evaluation of phenotype mapping coverage and unmapped terms. (A) Contribution of different NLP tools to total phenotype coverage. (B) Distribution of unique original terms mapped and unmapped across different data sources. (C) Top 30 most frequently unmapped traits, ranked by their occurrence. (D) Unique genes in mapped and unmapped parts. (E) Distribution of all mapped gene-phenotype associations by data sources. (F) Distribution of all mapped gene-SOC pairs, categorized by respective data sources.


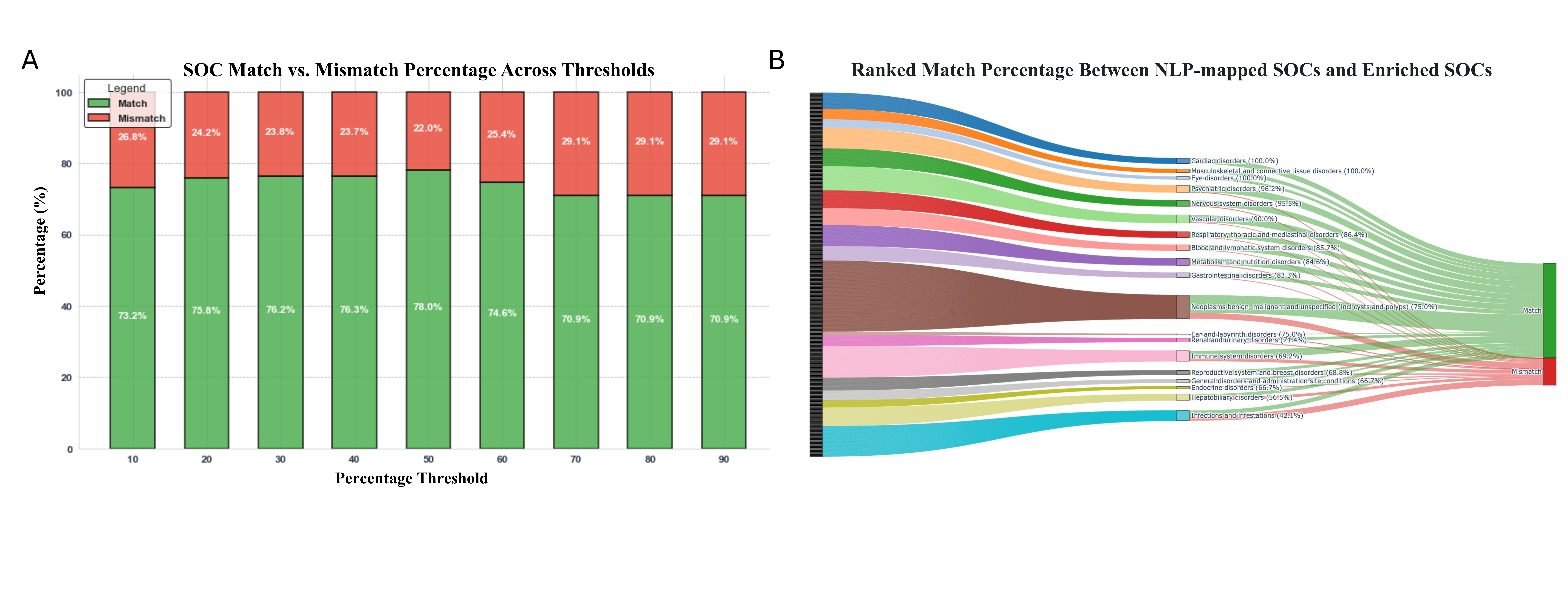
Supplementary Figure 3. Match rate across different cutoff thresholds and phenotype-to-SOC mapping comparison. (A) Match rate across different cutoff thresholds defining the SOC gene sets used for enrichment analysis. (B) Sankey diagram illustrating the mapping of phenotypes to enriched SOC categories, sorted by match percentage.





Supplementary Figure 4. Distribution of genes by cellular location and protein class. (A) Treemap visualization of gene counts categorized by cellular location. (B) Hierarchical diagram depicting gene counts by protein class. (C) Comparison of gene overlap between the old and new panels.


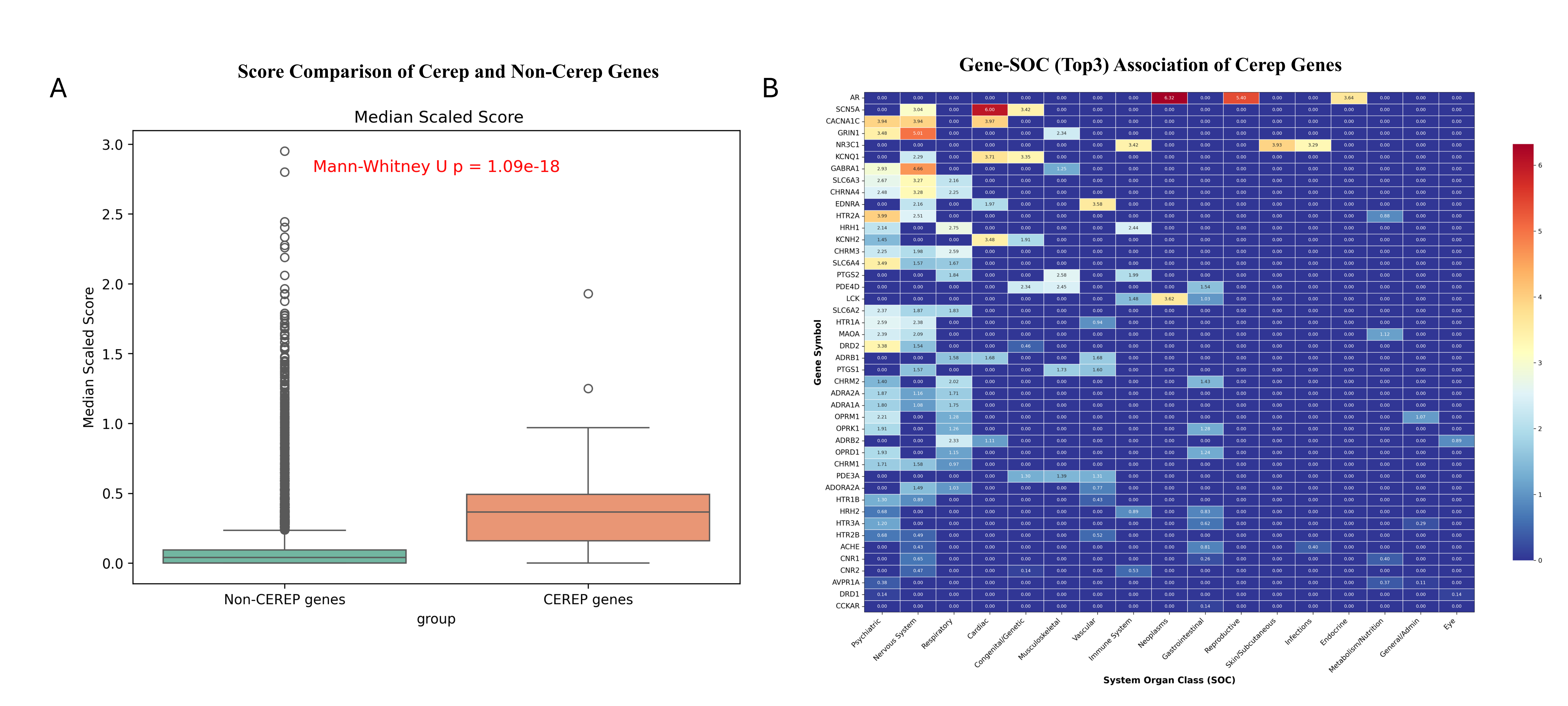


Supplementary Figure 5. Benchmarking the Safetyome against established secondary pharmacology targets (SafetyScreen44/CEREP). (A) Comparison of median scaled scores between SafetyScreen44/CEREP targets and non-panel genes. Boxplots summarize the distribution of gene-level median scaled scores, and statistical significance was assessed using a two-sided Mann–Whitney U test. (B) Heatmap of the top three associated SOCs for each target based on Safetyome. Color intensity reflects the scaled score, highlighting expected enrichment of nervous system/psychiatric and cardiovascular categories alongside additional system-specific signals.


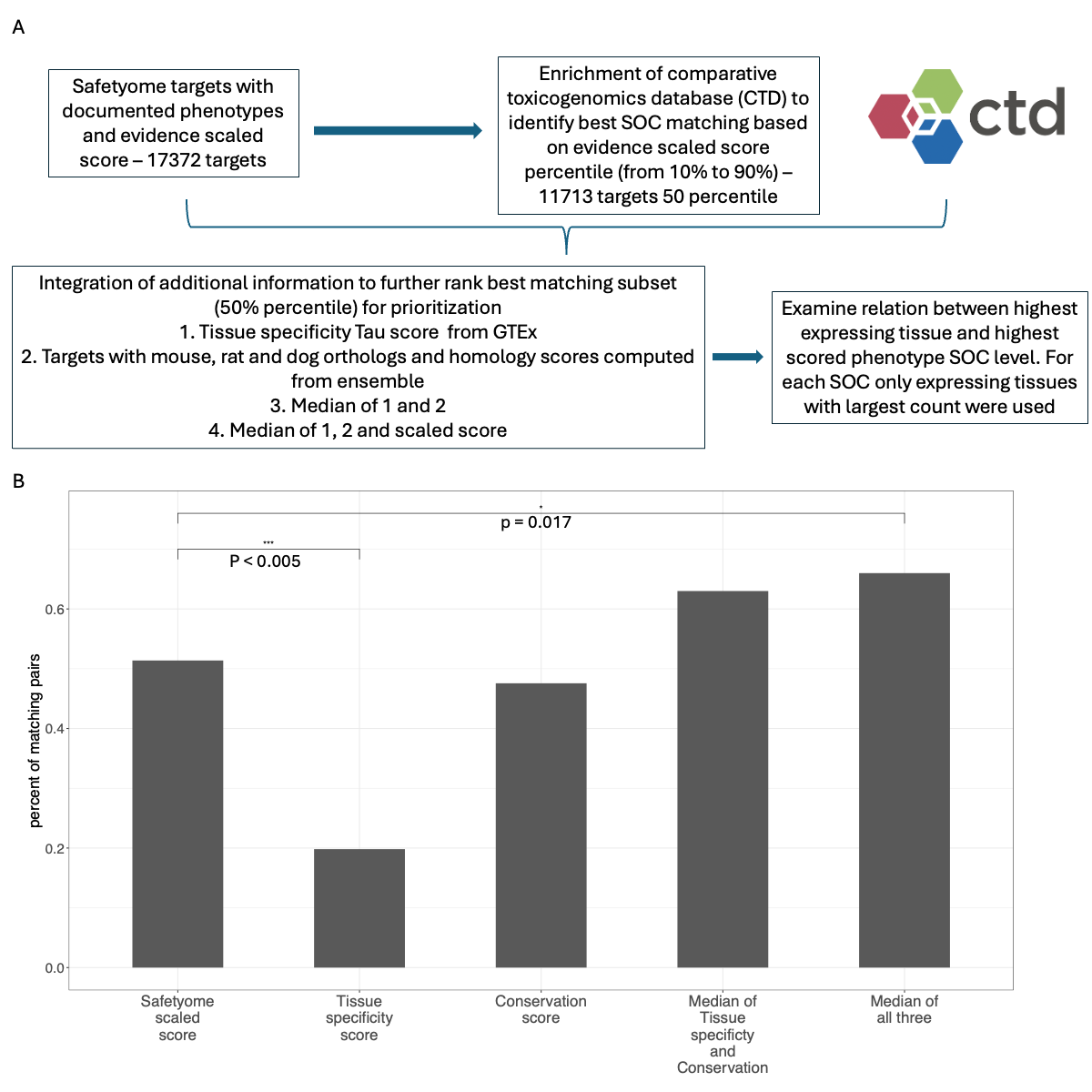


Supplementary figure 6. Prioritization of safetyome targets. (A) Flow chart of validation, subset selection and prioritization of safetyome targets (B) Barplot showing matching percentage of top 500 safetyome targets prioritized by different metrics with highest expressed tissues from GTEx. For each target only SOC level with highest scaled score were considered. For each SOC level only tissues with most SOC terms were included. Proportion test were used. * indicates pvalue < 0.05, ** indicates pvalue < 0.05 and *** indicates pvalue < 0.005


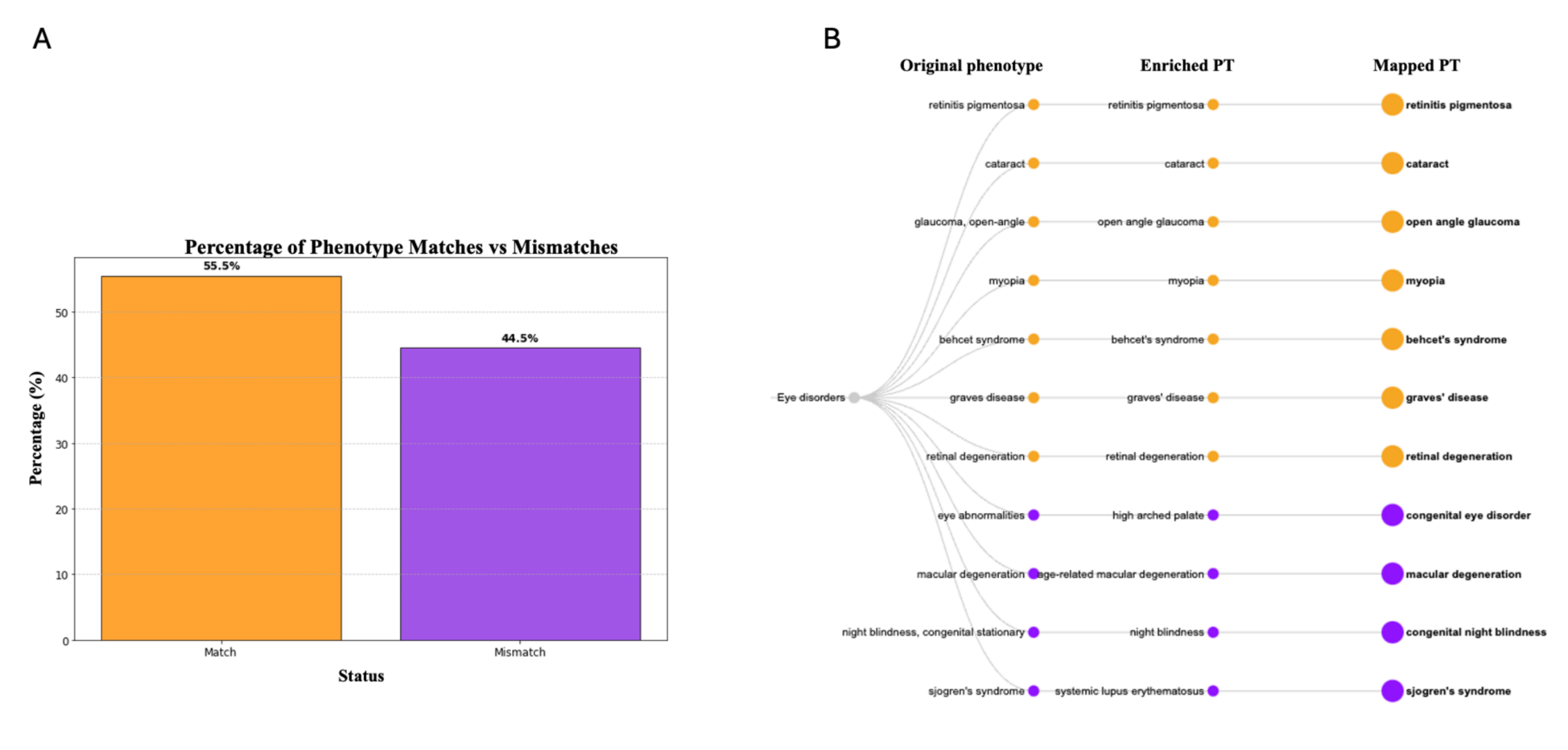


Supplementary Figure 7. Percentage of phenotype level match rate.
